# An Incidental Renal Oncocytoma: ^18^F-Choline PET/MRI

**DOI:** 10.3390/diagnostics6020014

**Published:** 2016-04-06

**Authors:** Andrew Mallia, Usman Bashir, James Stirling, Konrad Wolfe, Vicky Goh, Gary Cook

**Affiliations:** 1 Cancer Imaging Department, Division of Imaging Sciences and Biomedical Engineering, Kings College London, London SEI 7EH, UK; usman.bashir@kcl.ac.uk (U.B.); james.stirling@kcl.ac.uk (J.S.); vicky.goh@kcl.ac.uk (V.G.); gary.cook@kcl.ac.uk (G.C.); 2 Clinical PET Centre, St Thomas’ Hospital, London SEI 7EH, UK; 3 Department of Pathology, Southend University Hospital NHS Foundation Trust, SS0 0RY, UK; konrad.wolfe@southend.nhs.uk; 4 Department of Radiology, Guy’s & St Thomas’ Hospitals NHS Foundation Trust, London SEI 7EH, UK

**Keywords:** PET/MRI, ^18^F-Choline, oncocytoma

## Abstract

PET/MRI is a new hybrid imaging modality and has the potential to become a powerful imaging tool. It is currently one of the most active areas of research in diagnostic imaging. The characterisation of an incidental renal lesion can be difficult. In particular, the differentiation of an oncocytoma from other solid renal lesions such as renal cell carcinoma (RCC) represents a diagnostic challenge. We describe the detection of an incidental renal oncocytoma in a 79-year gentleman who underwent a re-staging ^18^F-Choline PET/MRI following a rise in PSA values (4.07, nadir 1.3).

**Figure 1:**
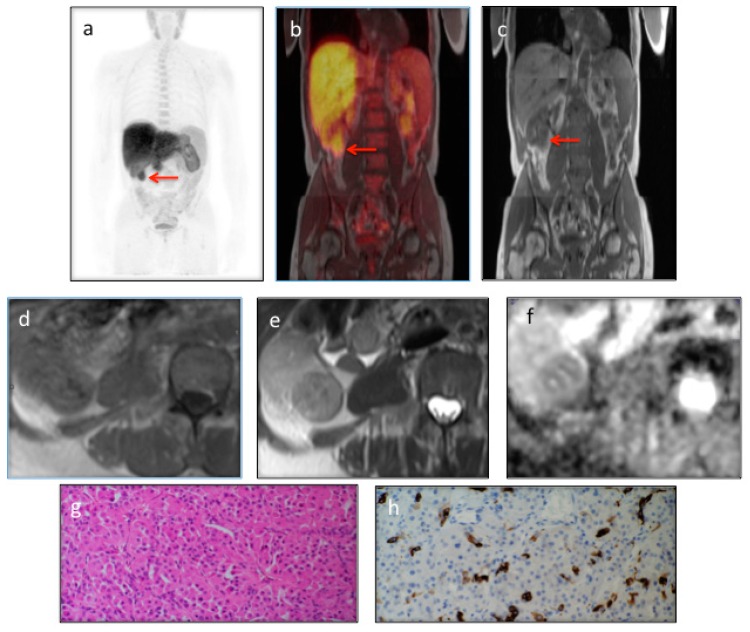
PET/MRI is a new hybrid imaging modality, which has recently been introduced into clinical practice [1]. We present a 79 year-old gentleman with a past history of prostate cancer Gleason 6 (3 + 3) treated with brachytherapy who underwent a re-staging ^18^F-Choline PET/MRI scan following a rise in PSA (4.07, nadir 1.3). Apart from an abnormal focus at the base of the left seminal vesicle, which was suspicious for disease recurrence, the ^18^F-Choline PET/MRI showed intense tracer uptake within a solid 34 mm right lower pole renal mass (**a**–**c**, **red** arrows), with MRI features typical for an oncocytoma: iso/hypo intense signal on T1-weighted (**d**); hyperintense on T2-weighted (**e**); and central area of low signal intensity on both T1-weighted and T2-weighted images suggestive of a central scar (**d**,**e**); The lesion appeared hindered on high *b* value (*b*-value = 900 s/mm^2^) diffusion weighted sequences (**f**). A right laparoscopic radical nephrectomy was performed subsequently. Macroscopically, a lower pole tan-coloured tumour was present. Sections of the tumour showed an encapsulated lesion comprised of packets of oncocytic cells staining positively for CK7, in keeping with an oncocytomas (**g** (200× magnification) and **h** (400× magnification)). The differentiation of an oncocytoma from other solid renal lesions, in particular renal cell carcinoma (RCC), is a diagnostic challenge [2]. MRI is frequently used for the detection and differentiation of renal masses, including oncocytomas. However the specificity of MRI is reduced since the features of oncocytomas show considerable similarities with those of RCCs [3]. Diffusion-weighted imaging (DWI) can augment MRI characterisation of renal lesions, but there is an overlap in the ADC values of RCCs and oncocytomas. Different authors have shown that malignant renal tumours have lower ADC values when compared to benign tumours such as oncocytomas [4]. However, other studies have demonstrated similar ADC values for these two tumour types [5]. PET/MRI maximises the diagnostic information available. This particular case demonstrated its value in the characterisation of an incidental renal lesion, thus avoiding the need for further diagnostic tests and potentially reducing unnecessary surgical resections. Choline-avid oncocytomas have not been described before, but cannot be differentiated from renal cell carcinomas on the PET characteristics alone. The addition of MRI features led to a confident PET/MRI diagnosis of an oncocytoma. All procedures performed in this study were in accordance with the ethical standards of the institutional and/or national research committee.
